# Retraction Note: Ultra-diluted *Toxicodendron pubescens* attenuates pro-inflammatory cytokines and ROS- mediated neuropathic pain in rats

**DOI:** 10.1038/s41598-019-44557-w

**Published:** 2019-06-11

**Authors:** Shital Magar, Deepika Nayak, Umesh B. Mahajan, Kalpesh R. Patil, Sachin D. Shinde, Sameer N. Goyal, Shivang Swaminarayan, Chandragouda R. Patil, Shreesh Ojha, Chanakya Nath Kundu

**Affiliations:** 1 Department of Pharmacology, R. C. Patel Institute of Pharmaceutical Education and Research, Shirpur- 425405, Dist. Dhule, Maharashtra India; 2 School of Biotechnology, Kalinga Institute of Industrial technology (a deemed to be University), Campus-11, Patia, Bhubaneswar, Odisha Pin-751024 India; 3 SVKM’s Institute of Pharmacy, Dhule-424001 Dist-Dhule, Maharashtra India; 4 Janmangal Homeopathy and Wellness Centre, Bopal, Ahmedabad, Gujarat 380058 India; 5 Department of Pharmacology & Therapeutics, College of Medicine & Health Sciences, UAE University, Al Ain, UAE

Retraction of: *Scientific Reports* 10.1038/s41598-018-31971-9, published online 10 September 2018

Following publication, the journal received criticisms regarding the rationale of this study and the plausibility of its central conclusions. Expert advice was obtained, and the following issues were determined to undermine confidence in the reliability of the study.

The *in vitro* model does not support the main conclusion of the paper that *Rhus Tox* reduces pain. The qualitative and quantitative composition of the *Rhus Tox* extract is unknown. Figures 1G and 1H are duplicates; and figures 1I and 1J are duplicates. The majority of experimental points reported in figure 3 panel A are duplicated in figure 3 panel B. The collection, description, analysis and presentation of the behavioural data in Figure 3 is inadequate and cannot be relied upon.

As a result the editors are retracting the Article. The authors do not agree with the retraction.

